# The role of postoperative radiotherapy in pN1 oral cavity cancer without extranodal extension

**DOI:** 10.1186/s12957-021-02396-y

**Published:** 2021-09-17

**Authors:** Tsung-You Tsai, Yenlin Huang, Andrea Iandelli, Shiao-Fwu Tai, Shao-Yu Hung, Huang-Kai Kao, Kai-Ping Chang

**Affiliations:** 1 Department of Otolaryngology-Head and Neck Surgery, Chang Gung Memorial Hospital, Kwei-Shan, Taoyuan, Taiwan; 2 Department of Pathology, Chang Gung Memorial Hospital, Kwei-Shan, Taoyuan, Taiwan; 3 Unit of Otorhinolaryngology and Head and Neck Surgery, IRCCS Ospedale Policlinico San Martino, Largo Rosanna Benzi, Genoa, Italy; 4 Department of Surgical Sciences and Integrated Diagnostics, University of Genoa, Viale Benedetto XV, Genoa, Italy; 5 Department of Plastic and Reconstructive Surgery, Chang Gung Memorial Hospital, Kwei-Shan, Taoyuan, Taiwan; 6 College of Medicine, Chang Gung University, Taoyuan, Taiwan

**Keywords:** N1, Oral cancer, Radiotherapy, Extranodal extension

## Abstract

**Background:**

The administration of postoperative radiotherapy remains controversial in pN1 oral cavity cancer patients without extranodal extension. The aim is to determine whether postoperative radiotherapy reduces the neck recurrence rate and improves the survival outcomes of pN1 patients.

**Methods:**

This study consecutively enrolled 1056 patients with newly diagnosed oral squamous cell carcinoma who underwent tumor wide excision and neck dissection from September 2002 to November 2019. One hundred two pN1 patients without extranodal extension were eligible for analysis. Then, a subgroup analysis of 40 patients was performed after patients with other adverse risk factors (positive margins, close margins, lymphovascular invasion, perineural invasion, tumor depth ≥ 10 mm, and poor histological differentiation) were excluded.

**Results:**

Of the 102 eligible pN1 patients, 26 patients received surgery alone, and 76 received postoperative radiotherapy. No significant differences were observed in the neck recurrence rate (7.7% vs. 15.8%, *p* = 0.30). Similarly, in patients without other adverse risk factors, no significant differences were observed in the neck recurrence rate (5% vs. 20%, *p* = 0.15) between surgery alone group and postoperative radiotherapy group. Moreover, no significant difference was found in the neck recurrence-free survival rate, overall survival, and disease-specific survival (77.1% vs. 52.5%, *p* = 0.42, 83.5% vs. 64.5%, *p* = 0.81, and 88.2% vs. 67.9%, *p* = 0.34, respectively).

**Conclusion:**

Postoperative radiotherapy did not significantly decrease the probability of neck recurrence and survival outcomes in pN1 patients without extranodal extension. Radical surgery alone may be considered sufficient treatment for pN1 patients without other adverse risk factors.

## Background

Oral cavity squamous cell carcinoma (OSCC) is one of the most common cancers worldwide, as there are 350,000 new cases per year, and it causes 180,000 deaths annually [1]. Although advances in surgical techniques and multidisciplinary treatment modalities have increased survival and quality of life of oral cancer patients in recent years, there are still many challenges associated with the treatment strategy due to a variety of clinicopathological manifestations of the tumor.

The presence of nodal metastasis may significantly affect the prognosis of OSCC patients, and regional recurrence is considered difficult to manage [2]. According to the American Society of Clinical Oncology practice guideline, postoperative radiation therapy (PORT) should be administered to patients with extranodal extension (ENE), multiple nodal metastasis, or contralateral node involvement (strength of recommendation: strong) [3–7]. However, whether PORT benefits pN1 patients without ENE or other adverse pathological features is still controversial. Although some studies have indicated that the regional recurrence rate is relatively low in pN1 OSCC patients, several comparative studies have claimed that PORT may benefit pN1 patients in terms of either survival outcome or regional recurrence rate [2, 8–13]. However, we should be aware that, in some of these studies, adverse risk factors that may confound the treatment outcomes, such as positive margins, perineural invasion, and depth of tumor invasion, were not recorded or analyzed in detail in the study design.

In the current study, we sought to investigate whether PORT could decrease the probability of developing regional recurrence or distant metastasis as well as survival outcomes in patients with pN1 status without ENE or any other adverse pathological factors. All adverse pathological factors were reviewed, and the potential benefit of PORT for pN1 OSCC patients was also assessed and analyzed after patients with all other adverse risk factors were excluded.

## Methods

Patients with OSCC who were treated at Chang Gung Memorial Hospital (Taoyuan, Taiwan) between September 2002 and November 2019 were consecutively enrolled and retrospectively reviewed. This study was approved by the institutional review board of our hospital. Previously untreated patients with primary OSCC tumors who underwent radical tumor excision and neck dissection were included. The exclusion criteria were as follows: prior history of any malignancy, pathologic ENE, confirmed distant metastasis or the development of a second primary cancer before surgery, and history of neoadjuvant radiation or chemotherapy therapy. The clinicopathological characteristics and survival outcomes of eligible patients with pN1 status without ENE were examined and analyzed (first group). Furthermore, we performed a subgroup analysis (second group) after patients with adverse risk factors other than pN1 and ENE status, according to the pathology reports, were excluded; these risk factors included positive margins, close surgical margins (< 5 mm), lymphovascular invasion, perineural invasion, depth of invasion ≥ 10 mm, and poor histological differentiation.

Preoperative assessment was performed for all patients and included a medical history review, physical examination, laboratory tests, computed tomography or magnetic resonance imaging, chest radiographs, bone scan or positron emission tomography, and liver ultrasonography. Primary tumors were radically excised, and primary closure or flap reconstructions, including local flap or microsurgical-free tissue transfer, were performed according to the defect size immediately after primary tumor ablation. Neck dissections, either selective or radical, were performed simultaneously. Intraoperative frozen sections were examined to confirm the adequacy of resection. Pathological staging was determined according to the 8th edition of the American Joint Committee on Cancer staging criteria [14]. The administration of adjuvant radiation therapy and chemotherapy was selected according to the NCCN guidelines [15]. Postoperative radiation therapy was considered if close (< 5 mm) margins, perineural invasion, bone invasion, advanced T stage (T3–T4) presented. Adjuvant chemoradiotherapy was considered if patients presented with positive margins, ENE, or pathologic multiple nodal metastasis. According to NCCN and our institutional guidelines, pN1 was not considered as an absolute indication for PORT in patients without other adverse risk factors [15]. Accordingly, in the second group, whether to receive PORT was sometimes optional for the patients. The prescribed dose was 2 Gy per fraction per day. The total radiation dose for patients was 60~70 Gy. The patients underwent regular follow-up visits every 2 months during the first year after discharge, every 3 months during the second and third year, and every 6 months thereafter. Follow-up image studies including ultrasonography, computed tomography, or magnetic resonance image were arranged regularly in an interval of 3–6 months for detecting possible recurrence or metastasis. Further biopsy or fine-needle aspiration would be performed if recurrence or metastasis was radiologically suspicious.

### Statistical analysis

Chi-square and Wilcoxon tests were used, as appropriate, to examine the differences in various clinicopathological features between the patient groups. Continuous data are presented as the means with standard deviations. Survival rates were evaluated using Kaplan-Meier plots and were tested using the log-rank test. All patients were followed-up until June 2020 or until death. Statistical analyses were performed using SAS software (version 9.3; SAS Institute, Cary, NC). All *p* values were two-sided, and a *p* value less than 0.05 indicated statistical significance.

## Results

### Patient demographics and clinicopathological characteristics

Between September 2002 and November 2019, 1056 patients with untreated and newly diagnosed OSCC were consecutively enrolled and retrospectively reviewed. In all, 102 eligible pN1 patients (91 males and 11 females) were included in this study. The median age of the sample was 55.1 years (range, 36.2–84.1 years). Table 1 contains the clinicopathological characteristics of these 102 subjects. Of these patients, 26 underwent surgery alone without PORT and 76 patients were treated with PORT. Unsurprisingly, pT status and overall pathological stage were more advanced in patients who received PORT (*p* < 0.001). Additionally, tumor depth was found to be higher in patients who were treated with PORT (*p* < 0.001). During follow-up, the neck recurrence rates were 7.7% (2/26) and 15.8% (12/76) in patients treated with surgery alone and with PORT (*p* = 0.30), respectively, while the distant metastasis rates were 3.8% (1/26) and 6.6% (5/76) in patients treated with surgery alone and with PORT (*p* = 0.61), respectively. The results demonstrated that observation or the administration of PORT did not appear to result in any differences in outcome with regard to neck control.

**Table 1:** Associations of adjuvant radiotherapy with clinicopathological characteristics—first group

	Adjuvant radiotherapy		
	(−)	(+)	*p* values
**Characteristics**	*n* = 26	*n* = 76	
**Age (years)**^**a**^	56.8 ± 10.9 (79.7, 41.0)	53.5 ± 9.0 (84.1, 36.2)	0.25
**Sex**			
Male	22	69	0.38
Female	4	7	
**Lesion site**			
Buccal mucosa	13	32	0.63
Mouth floor	3	4	
Gum	4	12	
Hard palate	0	2	
Lip	0	3	
Tongue	6	23	
**pT Status**			
1	3	6	< 0.001^*^
2	18	18	
3	2	18	
4	3	34	
**Overall pathological stage**			
III	24	40	< 0.001^*^
IV	2	36	
**Cell differentiation**^**b**^			
W-D+M-D	24	61	0.15
P-D	2	15	
**Tumor depth (mm)**^**a**^	7.9 ± 6.9 (30.0, 1.0)	14.1 ± 8.3 (34.0, 2.0)	< 0.001^*^
**Neck recurrence**			
(−)	24	64	0.30
(+)	2	12	
**Distant metastasis**			
(−)	25	71	0.61
(+)	1	5	

* Statistically significant

^a^Mean ± SD (maximum, minimum)

^b^*W*-*D* well-differentiated, *M*-*D* moderately differentiated, *P*-*D* poorly differentiated squamous cell carcinoma

Further analysis of the subjects in the study was performed after patients with adverse risk factors other than pN1 and pathologic ENE, including positive margins, close margins (< 5 mm), lymphovascular invasion, perineural invasion, depth ≥ 10 mm, and poor histological differentiation, were excluded. Forty patients with a median age of 56.1 (range, 36.6–79.8 years) were identified. Of the patients in this group, 20 were treated with PORT, while the other 20 were not treated with PORT. The clinicopathological characteristics are listed in Table 2. In this group, no significant difference was noted in the clinicopathological features including age, sex, tumor subsites, pT status, overall pathological stage, and American Society of Anesthesiologists physical status classification between the two subgroups (Table 2). The neck recurrence rates were 5% (1/20) and 20% (4/20) in patients who underwent surgery alone and those who were treated with PORT (*p* = 0.15), respectively. The distant metastasis rates were 0% (0/20) and 5% (1/20) in patients who were treated with surgery alone and those who were treated with PORT (*p* = 0.31), respectively.

**Table 2:** Associations of adjuvant radiotherapy with clinicopathological characteristics after excluding patients with adverse risk factors—second group

	Adjuvant radiotherapy		
	(−)	(+)	*p* values
**Characteristics**	*n* = 20	*n* = 20	
**Age (years)**^**a**^	55.7 ± 11.2 (79.7, 41.0)	56.4 ± 10.2 (72.3, 36.6)	0.56
**Sex**			
Male	17	19	0.29
Female	3	1	
**Lesion site**			
Buccal mucosa	10	8	0.47
Mouth floor	3	1	
Gum	2	2	
Lip	0	2	
Tongue	5	7	
**pT Status**			
1	2	3	0.24
2	15	9	
3	2	4	
4	1	4	
**Overall pathological stage**			
III	19	16	0.15
IV	1	4	
**ASA-PS classification**			
**1**	1	0	0.43
**2**	7	10	
**3**	12	10	
**Neck recurrence**			
(−)	19	16	0.15
(+)	1	4	
**Distant metastasis**			
(−)	20	19	0.31
(+)	0	1	

^a^Mean ± SD (maximum, minimum)

*ASA*-*PS* American Society of Anesthesiologists physical status classification

### Survival outcomes

**Fig. 1:**
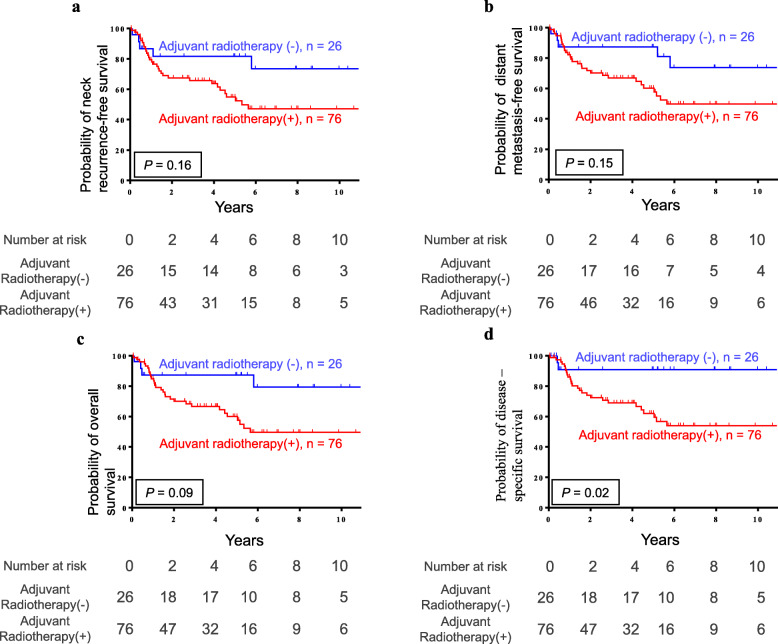
The Kaplan-Meier plots demonstrate the **a** neck recurrence-free survival, **b** distant metastasis-free survival, **c** overall survival, and **d** disease-specific survival in the 102 pN1 patients

The median follow-up time of the study participants was 4.3 ± 3.5 years, and the Kaplan-Meier survival curves for all subjects (102 patients) are presented in Fig. 1. The survival analysis was used to compare patients treated with surgery alone and those treated with PORT. The 5-year neck recurrence-free survival rate was 81.6% vs. 54.9% (*p* = 0.16), the distant metastasis-free survival was 87.2% vs. 60.1% (*p* = 0.15), the overall survival (OS) was 87.2% vs. 59.9% (*p* = 0.09), and the disease-specific survival (DSS) was 90.9% vs. 62.0% (*p* = 0.02) in patients who underwent observation alone and those who were treated with PORT, respectively.

**Fig. 2:**
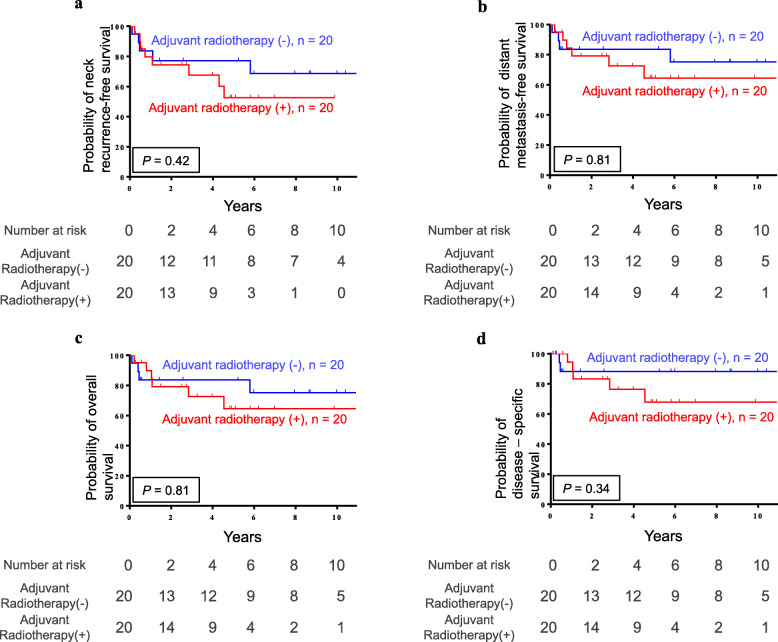
The Kaplan-Meier plots demonstrate the **a** neck recurrence-free survival, **b** distant metastasis-free survival, **c** overall survival, and **d** disease-specific survival in the 40 pN1 patients without other adverse clinicopathological features

The Kaplan-Meier survival curves of the 40 pN1 patients with no other adverse clinicopathological features, such as positive margins, close margins (< 5 mm), lymphovascular invasion, perineural invasion, depth ≥ 10 mm, and poor histological differentiation, are illustrated in Fig. 2. Similarly, no significant difference was found in the neck recurrence-free survival rate (77.1% vs. 52.5%, *p* = 0.42), distant metastasis-free survival (83.5% vs. 64.5%, *p* = 0.81), OS (83.5% vs. 64.5%, *p* = 0.81), and DSS (88.2% vs. 67.9%, *p* = 0.34) between patients treated with surgery alone and those treated with PORT.

## Discussion

Owing to the complex effects of multiple predisposing factors such as the habitual, environmental, and genetic factors on the OSCC carcinogenesis, the clinicopathological manifestation and treatment outcomes of OSCC patients varied significantly among different individuals [16–21]. New treatment strategies were proposed and adopted in attempt to optimize the treatment or functional results under some specific scenarios clinically [22–24]. The status of lymph node metastasis, which was known significantly associated with regional recurrence and survival, is clinically critical for deciding the indication of adjuvant radiotherapy or cheomoradiotherapy [25].

Whether pN1 OSCC patients without ENE or any other adverse pathological feature should be treated with PORT is still controversial and debated. Since no randomized controlled studies are available thus far to provide convincing evidence, PORT is advocated for pN1 patients with no other adverse pathological features in accordance with the practical experience of clinicians and the findings of retrospective studies. Unfortunately, the pN1 patients enrolled in these previous studies were usually quite heterogeneous and lacked consistent inclusion criteria, and thus, the results of these studies were not always comparative. Furthermore, without adequate controls for all the other adverse factors, the prognostic implications of PORT in pN1 patients without ENE could only be reported in terms of survival comparisons. Consequently, the potential benefits of PORT in pN1 patients without ENE and other risk factors seemed to be inconclusive among different studies.

A number of studies had investigated the benefit of PORT in pN1 patients; nonetheless, many of the them consisted of patients with positive ENE status or those with tumors in sites other than the oral cavity (elsewhere in the head and neck region) for which the treatment modalities or protocols could be completely different from those for OSCC [13, 26–28]. Previous studies that investigated the potential benefit of PORT in patients with pN1 OSCC without ENE were scarce and had many different limits. For example, Weiss et al. compared the 5-year OS, DSS, and recurrence-free survival in pN1 OSCC patients without ENE. However, in that study, the case number was limited, as only 18 OSCC pN1 patients were investigated, including 4 who received PORT and 14 who received surgery alone [29]. On the contrary, various endpoints were measured in different studies; however, neck recurrence rate, an important outcome for evaluating the benefit of PORT, was rarely reported in these prior studies. A lower regional recurrence rate in the PORT group was demonstrated in the study by Schiff et al., in which 40 pN1 patients with tongue cancer without ENE were analyzed. Nevertheless, all adverse pathological features except for ENE, including positive resection margins, were not documented and analyzed in this study. Although their impact on treatment outcome might not be as great as that of ENE or positive resection margins, other adverse pathologic features, such as perineural invasion, lymphovascular invasion, tumor depth, and close surgical margins, may still affect the prognosis after treatment and would therefore affect the study results. A few comparative studies controlled for these risk factors, but the inclusion/exclusion criteria were still incomplete and variable among studies. Feng et al. suggested that PORT may improve the DSS in pN1 patients with a lymph node yield less than 20, while Chen et al. reported a significantly better OS and disease-free survival of pN1 patients the PORT group [2, 30]. However, in these two studies, the enrollment of patients was confined to those with tongue cancer, and adverse pathological features, such as close margins and tumor depth, were not considered in these studies.

In single-center studies, the exclusion of adverse pathologic features other than pN1 and ENE may result in a smaller sample size. Thus, a larger data analysis of the National Cancer Database was conducted to assess the effect of PORT on pN1 patients. Chen et al., who identified 1467 pT1-2N1 OSCC patients, of whom 740 received PORT, indicated that PORT may be associated with improved OS [11]. Similarly, by analyzing a cohort of 1909 pN1 patients, Suresh et al. demonstrated that PORT conferred benefits in terms of OS [12]. However, like other studies that used a national database, detailed features and parameters are lacking in the analysis. In these studies, adverse risk factors, such as tumor depth, perineural invasion, or lymphovascular invasion, were not well controlled; additionally, more information about the study endpoint, such as locoregional recurrence, was also absent.

In the current study, OS, DSS, neck recurrence, and distant metastasis between the PORT and surgery alone groups were examined in two phases. In the first group, before adverse pathological features other than pN1 and ENE were excluded, PORT seemed to result in a notably worse outcome in terms of DSS (Fig. 1). This may be because the PORT group consisted of patients with more advanced pathological stage, higher cell differentiation, and advanced tumor depth (Table 1). According to prior research, poor tumor differentiation, close margins (< 5 mm), and tumor depth ≥ 10 mm were also independent risk factors for neck lymph node metastasis and clinical outcomes in OSCC patients [31–41]. To eliminate the confounding factors as much as possible, patients with adverse risk factors other than pN1 and ENE, including positive margins, lymphovascular invasion, perineural invasion, and especially, close margins (< 5 mm), depth ≥ 10 mm, and poor histological differentiation, were excluded from the second group. To the best of our knowledge, this is the first comparative study that simultaneously excluded tumor depth, poor histological differentiation, and close margins (< 5 mm) to investigate the benefit of PORT in pN1 OSCC patients. In contrast to most previous studies, PORT did not seem to confer any benefits with respect to OS, DSS, neck recurrence-free survival, and distant metastasis-free survival after all other adverse pathological features were excluded in pN1 OSCC patients without ENE (Fig. 2). The results of the current study implied that radical surgery alone may be considered an adequate treatment for pN1 patients with no other adverse risk factors.

In the current study, several limitations must be considered. Although the current study is a retrospective review of a large cohort, the number of patients for statistical analysis were relatively limited especially after we have excluded other adverse pathologic risk factors in the second group. Additionally, though the characteristics such as age, gender, staging, and tumor subsites were not significantly different between PORT and observation group, some confounding factors might still exist. Finally, because the administration of PORT was optional, potential selection bias might be unavoidable in the clinical scenarios. To clarify the effect of PORT in pN1 patients without other adverse factors pathologically with better statistical power, further studies with larger patient cohort or randomized prospective designs may be needed in the future.

## Conclusion

In pN1 OSCC patients without ENE, observation or the administration of PORT did not appear to result in differences in outcome with regard to neck control. Except for DSS, survival outcomes were not significantly different between the 2 groups. Even after patients with all other adverse risk factors such as positive margins, close margins (< 5 mm), lymphovascular invasion, perineural invasion, depth ≥ 10 mm, and poor histological differentiation were excluded, no significant difference was observed in the neck recurrence rate or in the distant metastasis rate. Moreover, OS, DSS, neck recurrence-free survival, and distant metastasis-free survival were not significantly different between patients treated with and without PORT. The results of the current study implied that radical surgery alone may be a sufficient treatment for pN1 patients with no other adverse risk factors.

## Data Availability

The datasets used and/or analyzed during the current study are available from the corresponding author upon reasonable request.

## Abbreviations

OSCC: Oral cavity squamous cell carcinoma
PORT: Postoperative radiation therapy
ENE: Extranodal extension
OS: Overall survival
DSS: Disease-specific survival
